# Medically-oriented design for explainable AI for stress prediction from physiological measurements

**DOI:** 10.1186/s12911-022-01772-2

**Published:** 2022-02-11

**Authors:** Dalia Jaber, Hazem Hajj, Fadi Maalouf, Wassim El-Hajj

**Affiliations:** 1 Electrical and Computer Engineering Department, American University of Beirut, Beirut, Lebanon; 2 Department of Psychiatry, American University of Beirut, Beirut, Lebanon; 3 Computer Science Department, American University of Beirut, Beirut, Lebanon; 4 Pathfinding, Automation Technology and Analytics, Intel Corporation, Hillsboro, Oregon USA

**Keywords:** Explainable models, Stress prediction

## Abstract

**Background:**

In the last decade, a lot of attention has been given to develop artificial intelligence (AI) solutions for mental health using machine learning. To build trust in AI applications, it is crucial for AI systems to provide for practitioners and patients the reasons behind the AI decisions. This is referred to as Explainable AI. While there has been significant progress in developing stress prediction models, little work has been done to develop explainable AI for mental health.

**Methods:**

In this work, we address this gap by designing an explanatory AI report for stress prediction from wearable sensors. Because medical practitioners and patients are likely to be familiar with blood test reports, we modeled the look and feel of the explanatory AI on those of a standard blood test report. The report includes stress prediction and the physiological signals related to stressful episodes. In addition to the new design for explaining AI in mental health, the work includes the following contributions: Methods to automatically generate different components of the report, an approach for evaluating and validating the accuracies of the explanations, and a collection of ground truth of relationships between physiological measurements and stress prediction.

**Results:**

Test results showed that the explanations were consistent with ground truth. The reference intervals for stress versus non-stress were quite distinctive with little variation. In addition to the quantitative evaluations, a qualitative survey, conducted by three expert psychiatrists confirmed the usefulness of the explanation report in understanding the different aspects of the AI system.

**Conclusion:**

In this work, we have provided a new design for explainable AI used in stress prediction based on physiological measurements. Based on the report, users and medical practitioners can determine what biological features have the most impact on the prediction of stress in addition to any health-related abnormalities. The effectiveness of the explainable AI report was evaluated using a quantitative and a qualitative assessment. The stress prediction accuracy was shown to be comparable to state-of-the-art. The contributions of each physiological signal to the stress prediction was shown to correlate with ground truth. In addition to these quantitative evaluations, a qualitative survey with psychiatrists confirmed the confidence and effectiveness of the explanation report in the stress made by the AI system. Future work includes the addition of more explanatory features related to other emotional states of the patient, such as sadness, relaxation, anxiousness, or happiness.

## Background

Although stress is a regular part of daily life, long-term stress can have severe consequences on health. Chronic mental stress can cause cardiovascular disease, depression, and increased susceptibility to infection [1]. The ability to detect when a person is stressed might therefore be very useful in the efforts to prevent health problems, especially in patients with suicidal thoughts [2]. Several artificial intelligence (AI) systems have been proposed for early automatic stress detection using physiological measurements such as electrocardiogram (ECG) and electromyography (EMG) taken from wearable devices [1, 3, 4]. The practical use of AI systems is limited, because people do not always trust the automated solutions. The primary reason for the lack of trust is a lack of transparent explanations of the results produced by AI models. Because the impact of wrong diagnosis is high, health professionals and patients are reluctant to adopt technologies that are not well understood. We are hence interested in developing an AI-based stress prediction model that automatically produces a report explaining the results of the AI evaluation in a way that is understandable and useful to human users. Understanding the reasons behind AI models’ predictions has become so crucial that the European Union developed new data privacy rules in 2018, where companies that use AI are obliged to provide either detailed explanations of individual AI algorithms or general information about how the algorithms make decisions when working with personal data [5].

Recently, there have been increasing efforts to develop explainable or interpretable AI systems, which make predictions and behave in ways that humans can understand [6]. Simple machine-learning (ML) models like decision trees, rule-based algorithms, and linear regression models may be considered interpretable, because they show the direct relationships between features and predictions. For more complex ML models, several approaches have been proposed to show the relationships, depending on the type of black-box model and the type of input data [6]. Some proposed approaches are model agnostic and can explain the outcome of any black-box model with any type of input [7, 8], whereas others focus specifically on deep neural networks used for image classification [9–11] or more general types of input [12].

**Fig. 1:**
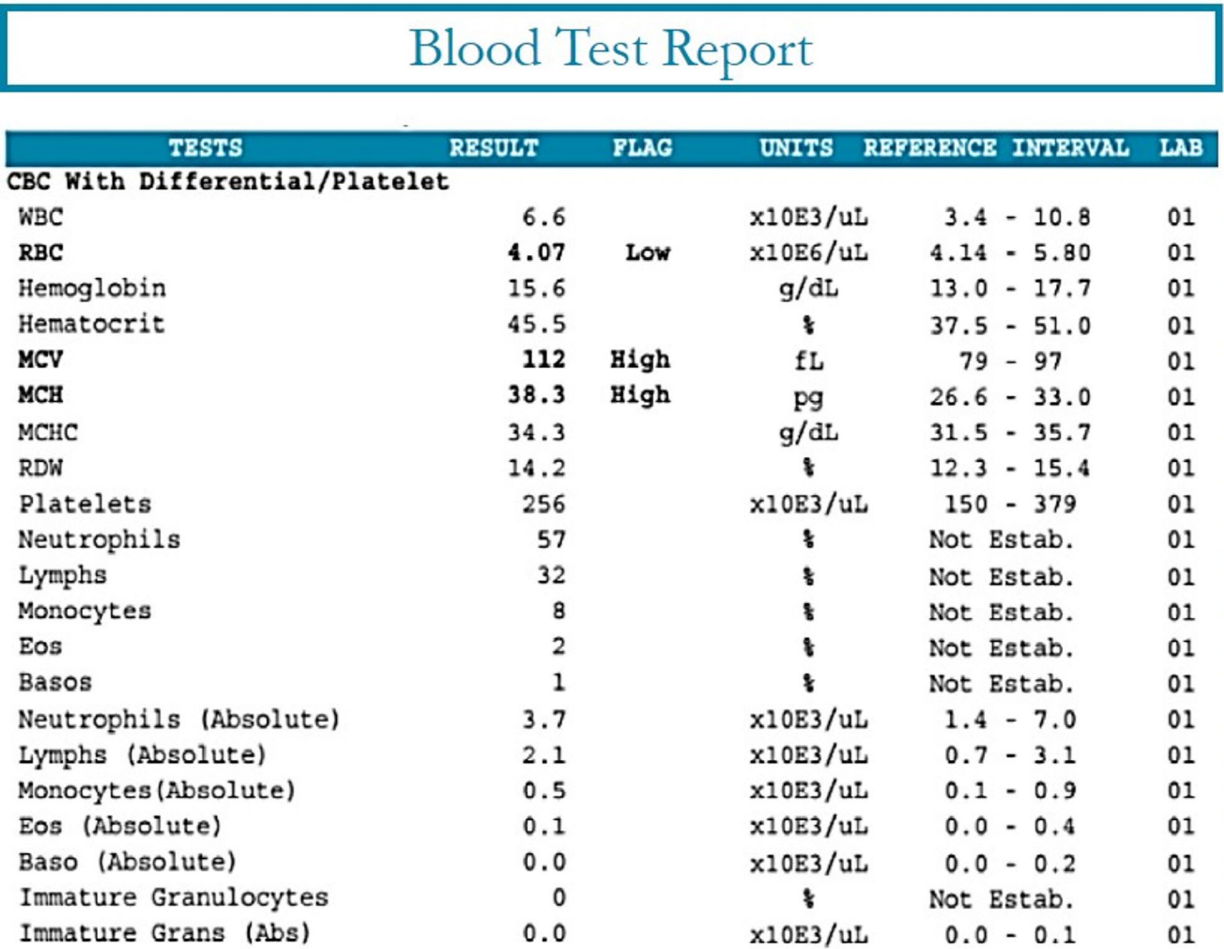
Sample of a blood test report [13]

In medicine, deep learning methods were used to create heat-maps to explain the predictions of AI systems that use medical images such as magnetic resonance images or X-ray images [14–16]. Other models were used to explain medical diagnoses by analyzing the influence of specific features on the diagnoses [16, 17]. However, no interpretable AI has yet been developed for stress prediction. One major limitation of previous interpretable AI approaches is that they fail to provide a user-centric explanation but instead focus on the mathematical relationships between features and predictions. Additionally, there are still no standard criteria by which to evaluate the interpretability of an AI system, nor is there even a clear definition of interpretability.

To address the lack of explainable AI systems for stress prediction, we propose a new design for an explainable AI system that predicts stress using data from wearable devices. The proposed explanatory component is inspired by medical blood test reports, which are already familiar to health care providers and patients. A sample of a typical blood test report is provided in Fig. 1. The stress explanation report includes the different physiological attributes that influence the overall probability that the subject is stressed and the reference ranges for each attribute. The explanation report and the blood test report share several look-and-feel aspects, including: the individuals features that are measured directly, the measured values of the features and the corresponding units, the range of normal values for the features, and flags that indicate any abnormal values. The abnormal values on the stress report are related to stress. In addition to those attributes, the stress report gives an overall probability of stress and a quantitative measure of the influence of each measured feature, referred to as the ‘IMPACT’, on the overall stress probability. We evaluated our proposed approach with a set of qualitative and quantitative experiments. The quantitative assessment focused on evaluating the different tests and features included in the report. The qualitative assessment was based on inputs from three expert psychiatrists to determine whether the report provides adequate explanation for the AI decisions.

In summary, the key contributions of our work are:

1. Design of an Explanation report for AI predicting stress. The design is medically oriented so that the report is familiar to health care providers and patients. The report includes stress prediction and the physiological signals related to stressful episodes, detailed in “Design of the explainable AI report” section.
2. Models and approaches for automatic generation of the report. The models include, not only the feature extraction and stress prediction, but also the model that derives the contribution of each physiological signal and the reference interval per vital sign. The different models are described in “Models for AI prediction and explanation” section.
3. Collection of Ground Truth from literature related to the physiological effect of stress. The ground truth data is used to provide information about how physiological features are affected by stress based on past scientific evidence. The collected data is listed in “Approach to extract the stress rangesand reference intervals” section.
4. Methods for quantitative and qualitative assessments to evaluate the effectiveness of the explainable AI report, explained in “Experiments and results” section.

The remainder of this paper is organized as follows. “Methods” section presents a literature review of existing explainable AI models and automated stress prediction systems. “Experiments and results” section covers our approach to design and produce the explainable AI report. “Discussion” section presents the evaluation of our approach. “Conclusion” section summarizes our findings and plans for future work.

### Related work

#### Explainable AI models

Approaches to make complex AI prediction models understandable to humans generally focus on clarifying the input-output relationship. Different approaches have been proposed for different types of data and prediction models. One important approach to attempt to explain any black-box model is the Additive Feature Attribution method, in which the original black-box model is approximated with a simpler model that is easily explainable. The approximation is composed of a linear combination of binary variables, as shown in Eq. 1

1 $$\begin{aligned} g(z^{'}) =\phi +\sum _{i=1}\phi _{i}z_{i}^{'} \end{aligned}$$

where $z^{'} \in [0,1]^{M}$, with M as the number of simplified input features; and $\phi _{i} \in {\mathbb {R}}$, which represents the contribution of feature $z_i$ to the model’s prediction. In the simplified features vector, a feature with a value of ’1’ is present in the subject, and a feature with a value of ’0’ is absent in the subject. Another approach that is commonly used to explain black-box models is local interpretable model-agnostic explanations (LIME) [7]. In the LIME approach, the input data are perturbed, and the effects of the perturbation on the output are assessed. LIME then tries to approximate the machine learning (ML) model with another model that is easily interpretable. The interpretable model is a linear combination of the input variables with some simplifications and perturbations. The LIME model presents as an output a list of explanations, reflecting the contributions of each variable to the results of the original ML model. A weak point of the LIME approach is the instability of the explanations, which can differ greatly with small changes in the input data.

The shapley additive explanations (SHAP) approach [8] combines LIME with Shapely values [18], a concept in cooperative game theory that was developed to distribute the gains from a cooperative game to players, or features. SHAP uses locality approximation and Shapely additive values to provide an explanation for any black-box model. The method uses three criteria: local accuracy; missingness, which does not give any importance to missing features; and consistency, which makes sure that even if a model changes, the feature impact will still have the same attribution assigned. To interpret the prediction of a convolutional neural network (CNN), Zhou et al. [9] introduced the concept of class activation mapping (CAM), which indicates the discriminative image regions used by the CNN that impact target classification. CAM only works on CNNs that are composed of a global average pooling (GAP) layer preceding a fully connected layer that produces the output. Deep learning important features (DeepLIFT) [12] is another approach that uses back-propagation to explain a CNN model. DeepLIFT decomposes the output of a neural network for a specific input by back-propagating the contribution of every feature of the input. The layer-wise relevance propagation (LRP) [11] method is equivalent to DeepLIFT with the reference activation of all neurons set to zero. The main idea behind the LRP algorithm is to explain a classifier’s prediction specific to a given data point by using the topology of the learned model to attribute relevance scores to components of the input.

In healthcare, explainability is important since decisions made by ML models can have an impact on the patients’ safety [19]. In this domain, explainable AI applications have been developed to interpret data from imaging studies. A recent study to detect COVID-19 using chest X-ray images [14] introduced a technique called GSInquire that created heatmaps to confirm the diagnostic features learned by the proposed COVID-net model. To study the reliability of a CNN model designed to identify brain tumors in MRI images, Pereira et al. [15] used GradCAM, an improvement of CAM, to create heat-maps that show the factors that influenced the classification of features as tumors. For computed tomography (CT) imaging, a sensitivity analysis was applied to liver CT images to explain the segmentation of tumors [16]. The analysis was performed by maximizing the target neuron using gradient ascent. Another new ML system called Prescience was introduced [17] to interpret real-time predictions to prevent hypoxemia during surgery. The Prescience model uses SHAP attribution to analyze preoperative factors and in-surgery parameters. In another study [20], a framework was proposed for the design of an explanatory display to interpret the prediction of a pediatric intensive care unit in-hospital mortality risk model. The explanation was displayed in a user-centric manner and established using Shapely values. Explainable models have not been applied to stress prediction based on physiological sensor data. Explainable AI systems for stress prediction need to augment their explanations with additional predictive models that provide descriptions of biological factors other than the stress state per se.

#### Stress prediction systems

There have been several attempts to create automatic stress prediction systems, each using different features to predict or detect stress. To reduce privacy concerns and power consumption, some approaches only use data from accelerometers. For example, Garcia-Ceja et al. [21] extracted 34 features from the time and frequency domains of accelerometer data and fed them into several classification models including Naives Bayes, decision tree, and random forest. They were able to achieve an accuracy of 71% using decision trees. In addition to accelerometer data, Giakoumis et al. [22] included galvanic skin response (GSR) and electrocardiogram (ECG) data and behavioral features to predict stress and found that prediction based on the physiological data and the behavioral features was more accurate than prediction based on physiological data alone. Sun et al. [23] were able to obtain an overall accuracy of 92.4% for 10-fold cross validation using GSR, ECG, and accelerometer data. Carneiro et al. [24] added a video camera and pressure-sensitive touchscreens to accelerometers and obtained an accuracy of 78% in classifying touches as stressed or not stressed using J48 tree. Bomogolov et al. [25] predicted stress with 72.39% accuracy using a random forest classifier based entirely on call logs, Bluetooth data, and short message service (SMS) data from users’ mobile phones. When those data were combined with global positioning system (GPS) and Wi-Fi information, these features used allowed to detect a change of behavior in about 86% of the participants during stressful times [26]. Li et al. [27] implemented a deep neural network model to perform two classification tasks. A binary stress detection and a 3-class emotion classification using physiological signals collected from wrist-worn and chest-worn sensors. They were able to achieve high prediction accuracy of around 99% for both tasks. A summary of literature on stress prediction systems is presented in Table 1, listing the different measurements and models used per experiment as well as the highest accuracy obtained.

**Table 1:** Summary of literature review on stress prediction systems

Measurements	Prediction model	Stress prediction accuracy	Paper
Accelerometer, 34 features from the time and frequency domains of accelerometer data	Naives Bayes, Decision Trees, and Random Forest Classifiers	Highest accuracy 71% using decision trees	[21]
Accelerometer, GSR, ECG and behavioral features	LDA (Linear Discriminant Analysis)-based classifier	Prediction based on the physiological data and the behavioral features was more accurate than prediction based on physiological data alone	[22]
Accelerometer, GSR, ECG	Decision Tree Classifier	92.4% for 10-fold cross validation	[23]
Accelerometer, video camera, pressure-sensitive touchscreens	J48 tree	78% in classifying touches as stressed versus not stressed	[24]
Call logs, Bluetooth data, and SMS data from users’ mobile phones	Random Forest Classifier	72.39% for binary classification, stressed versus not stressed	[25]
Physiological data collected from chest-worn and wrist-worn sensors	Deep Convolutional Neural Network	99.80% accuracy rates for binary classification for stress detection	[27]

Although stress detection has been widely studied, it is still challenging to explain the results of the detection systems in a way that is easily understandable to humans. It is important for health care professionals and patients to understand the reasons behind decisions made by AI models, because the impacts of those decisions can be serious. Many of the models described in the literature to predict mental stress use complex algorithms to achieve accurate predictions; however, the interpretability of the models tends to decrease as the accuracy increases. Hence, there is a need for models that provide explanations and interpretations for complex stress prediction.

## Methods

### Problem description and objectives

The objective of this work is to provide an explanation of the stress prediction conducted by AI systems that take as input the physiological signals listed in Table 2. The generated explanations need to be physician and patient friendly.

**Table 2:** Physiological Measurements

Signal	Measurement
Electrocardiogram (ECG)	Electrical activity of the heart
Electromyography (EMG)	Electrical activity of muscles at rest and during contraction
Electrodermal activity (EDA)	Wrist and chest skin conductance
Temperature	Wrist Temperature
Respiration	Respiration rate and cycle

**Fig. 2:**
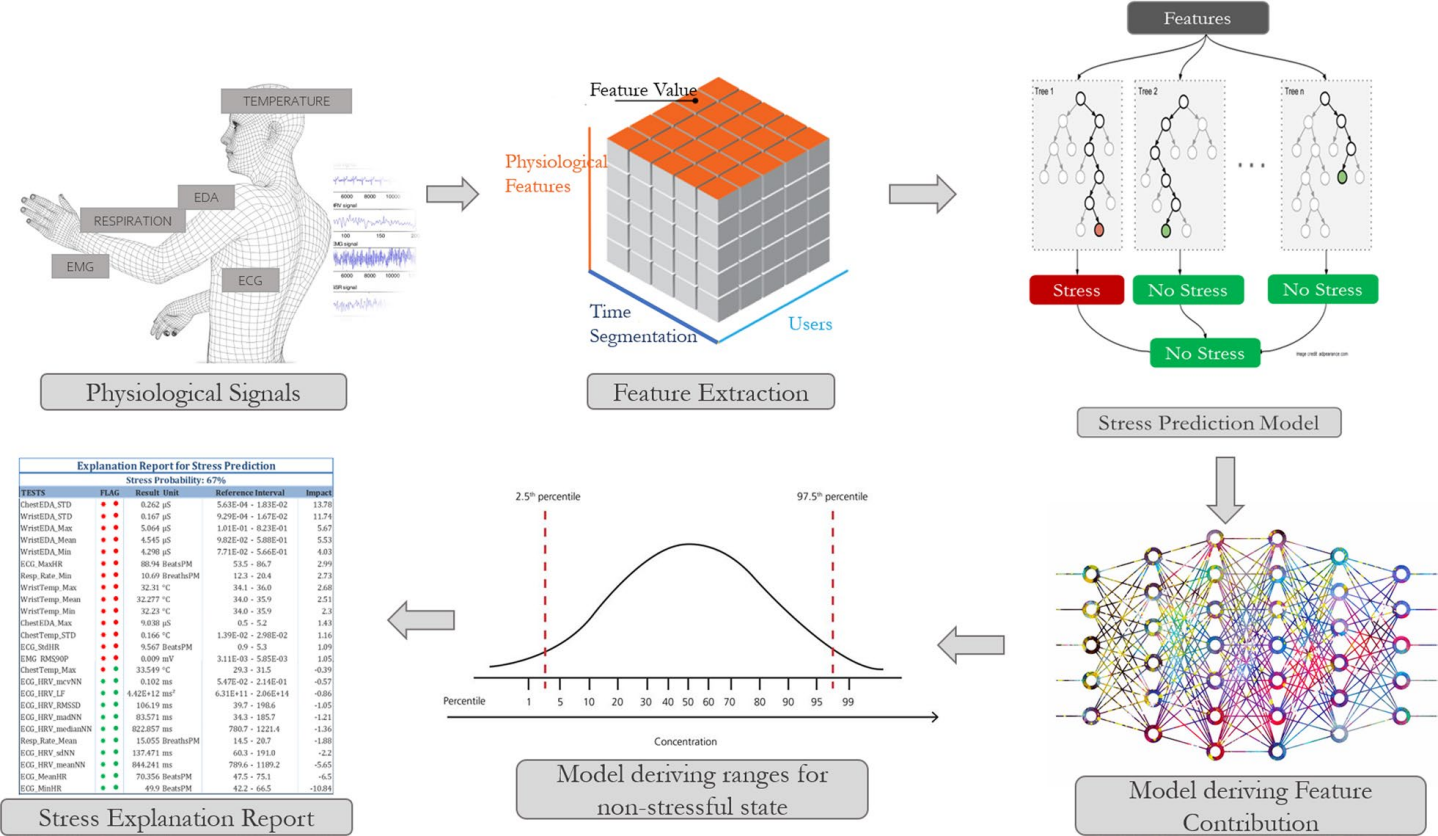
The proposed solution

There are several challenges that we aim to address. The first challenge is to determine what explanation should be displayed for physicians and patients and how the explanation should be presented. The second challenge is to develop models that can produce the necessary explanations. In order to produce the desired explanations, three models are needed as shown in Fig. 2. The first model extracts the desired physiological features by applying statistical signal processing to physiological data from ECG, EDA, EMG, respiration, and temperature sensors. The second model derives the contribution of each feature to the overall stress prediction using a separate, feature-based classifier that takes as input the pre-processed features. The third model determines the ranges of feature values that are indicative of a non-stressful state.

### Design of the explainable AI report

#### Proposed explanations and corresponding user interface

Inspired by standard reports of blood test results, we propose to have the AI system automatically generate a report showing the measured values and normal ranges for each component of the stress assessment. The aim is to help patients and health care professionals understand which physiological factors are related to stressful episodes experienced by the patients. For the collection of the measurements needed to generate the explainable AI report, the patient needs to stay still for a maximum of 3 min with a set of sensors. Even though only a 90-second interval is needed to extract the physiological measurements, the additional time is recommended taking into consideration any faulty data.

#### Layout of the report

For ease of reference, a sample blood test report is shown in Fig. 1. The key aspects of the blood test report include:

- TESTS: the different blood tests included in the report
- RESULT: the measured values of the different blood tests
- FLAG: indicators of normal/abnormal test results
- UNITS: the units of the measured values
- REFERENCE INTERVAL: the range of normal test values

**Fig. 3:**
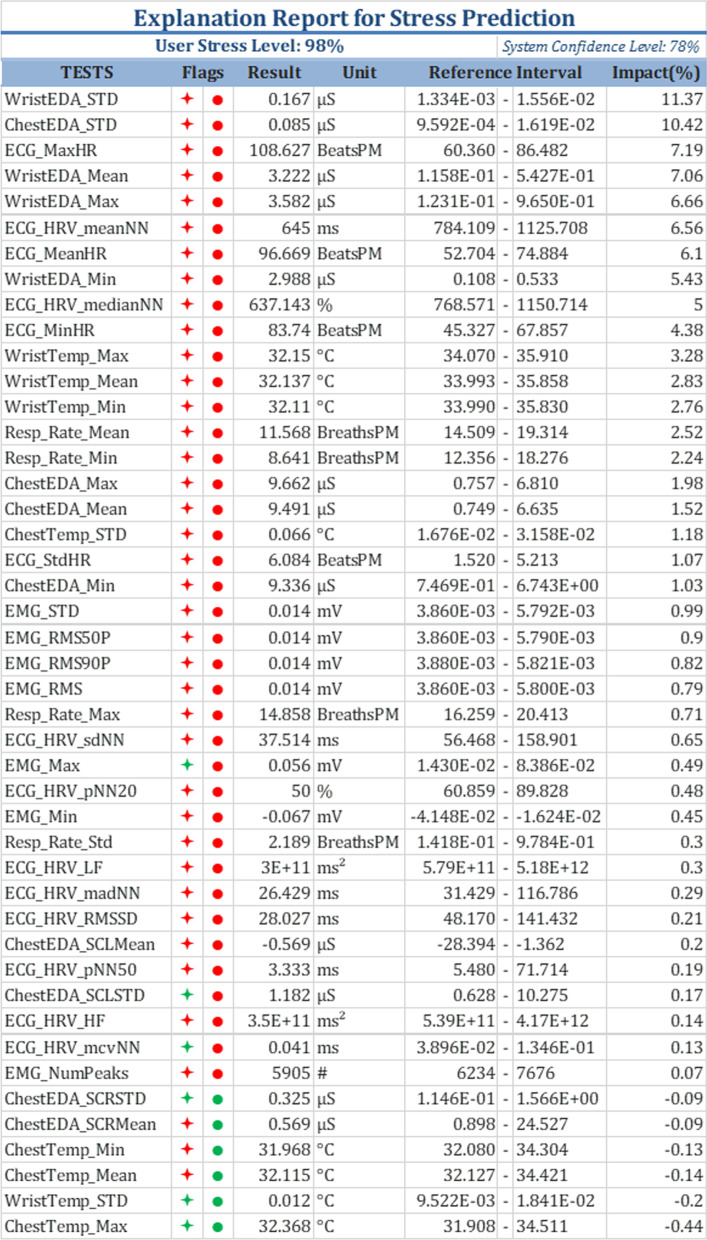
An example of a stress prediction report

To make our AI-generated stress prediction report compatible with what patients and health care professionals are used to seeing, we will use a similar organization. An example of how the stress prediction report will look is shown in Fig. 3. This report will include:

- USER STRESS LEVEL: the stress level of the patient in percentage, varying between ‘Not stressed’ (0%) and ‘Extremely stressed’ (100%).
- SYSTEM CONFIDENCE LEVEL: Accuracy of the stress prediction model which can be considered as a historic accuracy based on historic data.
- TESTS: the different physiological signals included in the report, extracted from the signals listed in Table 3.
- RESULT: the measured values of the physiological signals, typically presented as statistical measures (e.g., mean or median over a given interval) of the raw data.
- UNIT: the units of the measured values.
- REFERENCE INTERVAL: the range of normal values for the physiological signals under non-stressful conditions. (“Derivation of the stress ranges and reference intervals” section shows how the intervals are derived).
- IMPACT: the percentage contribution of each physiological signal to the overall stress prediction (“Model to derive the contributions of each feature to the stress prediction” section explains how the impact is calculated).
- FLAGS: indicators of normal/abnormal physiological signals. Red indicates values associated with stress, whereas green indicates values not associated with stress. Star-shaped flags represent correspondence to the REFERENCE INTERVAL. Circle-shaped flags represent the IMPACT of the test result on the overall prediction. Therefore a red star-shaped flag indicates that the test result is outside of the reference interval, as for the red circle-shaped it indicates that the IMPACT of the test result is positive.

We also introduce an online analytical processing (OLAP) Customization approach. Our stress evaluation report allows for different levels of customization that are common with decision support systems. The detailed list of physiological measurements can be treated as a multi-dimensional OLAP data warehouse. Different levels of extracts and aggregations can be generated and customized to fit users’ needs. For example, a simple aggregate custom report might include only heart rate, respiration, and body temperature.

#### Choice of TEST signals

The physiological measurements included in the report are commonly used in experimental procedures to study the biological effects of stress [28]. Additional features that are crucial to the explanation of the stress prediction are shown in Table 3. Acerbi et al. extracted several EDA and ECG features and reported the values at baseline and during stress [29]. They then performed t-tests to identify features which values differed between stressful and non-stressful conditions. In another study, the same procedure was followed using only EMG signals [30]. For the temperature and respiration features, statistical measures are extracted including the mean, maximum, minimum, and standard deviation.

#### Choice of dataset

The wearable stress and affect detection (WESAD) dataset [28] consists of different physiological measurements recorded during stressful and relaxed conditions. It contains physiological and motion data recorded from wrist-worn and chest-worn devices. The devices used are the RespiBAN Professional,^1^ which is placed around the subject’s chest, and the Empatica E4,^2^ which is worn on the subject’s non-dominant hand. The modalities include EDA and temperature data from an Empatica C4 device. The RespiBAN device provides data on respiration; ECG; EDA recorded on the rectus abdominis, considering that the abdomen has a high density of sweat glands; EMG recorded on the upper trapezius muscle on both sides of the spine; and temperature recorded on a sensor placed on the sternum.

Data were collected from 15 graduate students in a laboratory setting. Each subject experienced three conditions:

1. *Baseline* Users were provided neutral reading material (e.g., magazines).
2. *Amusement* Users watched a set of funny videos.
3. *Stress* Users were exposed to the Trier Social Stress (TSST), which is used to induce stress in participants. The TSST generally includes three phases: an anticipatory speech preparation, speech performance, and verbal arithmetic.

From this dataset we extract the TEST signals specified in “Choice of TEST signals” section.

**Table 3:** Stress explanation features

Signal	Features	Description
ECG	$\mu _{HR}, \sigma _{HR}$	Mean, standard deviation,
	$Max_{HR},Min_{HR}$	Maximum and minimum heart rate (bpm)
	$HF_{HRV}$	Variance in HRV in the high frequency range (.15–.40 Hz)
	$LF_{HRV}$	Variance in HRV in the low frequency range (.04–.15 Hz)
	$\vert \mu \vert _{NN}, \sigma _{NN},Mad_{NN}$	Mean of the absolute values, standard deviation, median absolute deviation
	$Med_{NN},MCV_{NN}$	Median, and median-based coefficient of variation of the successive differences between the RR intervals
	$RMSSD_{NN}$	Root mean square(RMS) of the RR interval
	$PNN_{20}$, $PNN_{50}$	Number of interval differences of successive RR intervals greater than 20 ms or greater than 50 ms
EMG	$\mu _{EMG}, \sigma _{EMG}$	Mean, standard deviation, maximum, and minimum
	$Max_{EMG}, Min_{EMG}$	Values of EMG activity in the lower trapezius
	$Peaks_{EMG},RMS_{EMG}$	Number of peaks in signal, normalized RMS value
	$RMS50P_{EMG},RMS90P_{EMG}$	50th, 90th percentile of rank-ordered RMS values
EDA	$\mu _{WristEDA},\sigma _{WristEDA}$	Mean, standard deviation, maximum, and minimum
	$Max_{WristEDA}, Min_{WristEDA}$	Values of EDA connected to the user’s wrist
	$\mu _{ChestEDA},\sigma _{ChestEDA}$	Mean, standard deviation, maximum, and minimum
	$Max_{ChestEDA}, Min_{ChestEDA}$	Values of EDA connected to the user’s chest
	$\mu _{ChestSCL},\sigma _{ChestSCL}$	Means and standard deviations of the skin
	$\mu _{ChestSCR},\sigma _{ChestSCR}$	Onductance level and skin conductance response
Respiration	$\mu _{RespRate},\sigma _{RespRate}$	Mean, standard deviation, maximum
	$Max_{RespRate}, Min_{RespRate}$	and minimum of the respiration rate
Temperature	$\mu _{WristTemp},\sigma _{WristTemp}$	Mean, standard deviation,
	$Max_{WristTemp}, Min_{WristTemp}$	Maximum and minimum values of the temperature measured from the user’s wrist

**Table 4:** ECG features shown experimentally to indicate stress [29]

Physiological feature	Range for no stress	Range for stress	*p* Value
$\mu _{NN}(ms)$	788$\pm$ 126	642$\pm$ 96	0.005
$\mu _{HR}(BPM)$	78.45$\pm$ 12.38	95.54$\pm$ 13.69	0.005
$\sigma _{HR}(BPM)$	6.43$\pm$ 1.15	10.48$\pm$ 3.88	0.001
$RMSSD_{HRV}(s)$	0.04$\pm$ 0.02	0.03$\pm$ 0.01	0.018
$pNN50_{HRV}(s)$)	22.89$\pm$ 19.44	7.35$\pm$ 4.98	0.043

### Models for AI prediction and explanation

#### Model to derive the contributions of each feature to the stress prediction

An important aspect of the stress evaluation report is the IMPACT, or indication of how much each factor contributes to the overall stress probability. To calculate the impact for each factor, we customized the SHAP model, where the total probability of stress $P_{X}(Stress)$ for each set of TEST measurements *X* is computed as the sum of the mean probability $P_{Avg}(Stress)$ and the individual contributions of each TEST feature as seen in Eq. 2.

2 $$\begin{aligned} P_{X}(Stress) =P_{Avg}(Stress)+\sum _{i \in F_{1}, \ldots ,F_{N}}^{} \phi _{i} \end{aligned}$$

where *F* represents the choice of physiological feature, and *N* represents the number of features for observation *X*. $P_{Avg}(Stress)$ represents the probability of a random person being stressed. The $\phi _{i}$ , also known as the SHAP value, is used to derive the percentage contribution of each feature. A positive value indicates that the feature reinforces the prediction of stress, whereas a negative value indicates a negative contribution, which is an indication of non-stress. Those contributions indicate deviation from the average probability of stress $P_{Avg}(Stress)$ .

The SHAP $\phi _{i}$ values for each feature *i* can be calculated using any ML classifier by removing (nullifying) the features *i* one at a time and then computing the resulting predictions. In our model, we used a random forest classifier. Mathematically, the $\phi _{i}$ is computed based on Eq. 3.

3 $$\begin{aligned} \phi _{i}=\sum \big [ {f}_{{(S}{ \bigcup } \{i}{)}{(}x_{{(S}{\bigcup }\{i}{)}{)-(}f_s{(}x_s{)\big ]} \left( \frac{ \vert S \vert ! ( \vert M \vert - \vert S \vert -1 ) !}{ \vert M \vert !}\right) \end{aligned}$$

where *S *is a set of indexes in z’ (as seen in Eq. 1), M is the set of all input features, $x_S$ represents the values of the input features in the set S, and $f_{()}$ represents the hypothesis function for the classifier. To obtain the SHAP values, a model $f_{s}$ is trained with the feature *i* withheld, and another model $f_{( S \bigcup {i})}$ is trained with that feature present. Then, the predicted values from both models are compared to the current input $x_S$.

The IMPACT measure is calculated as the percentage of the features’ contributions $\phi _{i}$ as follows:

4 $$\begin{aligned} IMPACT_{i,X} (\%) =\left( \frac{\phi _{( i,X)}}{\sum _{Features} \vert \phi _{X} \vert }\right) \end{aligned}$$

The $P_{Avg}(Stress)$ can be computed from historical training data by computing the percentage of individuals who are stressed, or the average of the stress probability:

5 $$\begin{aligned} P_{Avg}(Stress) = Mean(y_{train}) \end{aligned}$$

where $y_{train}$ represents true labels of stress predictions for individuals available in historical training data.

The authors of SHAP also proposed KernelSHAP and TreeSHAP and provided many global interpretation methods. KernelSHAP is an approach to estimate Shapely values inspired by local surrogate models, which are interpretable models used to explain the predictions of any black-box ML model. With KernelSHAP, it will be possible to use any classification model to provide the stress prediction. As for the TreeSHAP, it provides interpretation for any tree-based model and has a faster implementation than KernelSHAP. TreeSHAP reduces the computational complexity from $O(TL2^{M})$ , the complexity in KernelSHAP, to $O(TLD^{2})$ , where T is the number of trees, L is the maximum number of leaves in any tree, and is D the maximal depth of any tree. In addition to being computationally faster, TreeSHAP allows the creation of different visualizations that can help users understand the interpretation. Therefore, we used TreeSHAP as the model that assigns the feature contribution.

#### Random forest classifier for stress prediction

The measurements in the RESULTS column of the stress evaluation report are used as inputs to the stress prediction model, which indicates if the user is stressed or not stressed according to each measurement. TreeSHAP requires the prediction model to be a tree-based model. Driven by the fact it provides solid prediction results and works well with imbalanced dataset, the balanced random forest classifier was our choice for stress prediction. The random forest is an ensemble method used for classification or regression. It is trained using a bagging method, which consists of randomly selecting a subset of the training set, fitting a decision tree to each subset, and finally combining the results. For classification, the random forest uses the majority votes for the class prediction; because each tree provides one vote, the final vote can be the mode or the most frequent class predicted by each tree. When working with an imbalanced dataset, a version of the random forest classifier known as the ‘balanced random forest’ is highly useful. The balanced random forest model randomly under-samples each bootstrap sample to balance the labels. The data was split into 90% for training, 10% for testing, and 10% validation, separated by users. For the evaluation, a leave-one-user-out cross-validation scheme is employed where the data of one user are held out for testing while the data of the rest of the users are used for training. Hyper-parameter optimization techniques are implemented to fine-tune the stress prediction model. We use, from the scikit-learn library, “GridSearchCV” to select the optimal random forest hyper-parameters.

### Approach to extract the stress ranges and reference intervals

The following section describes how we generate stress ranges and reference intervals using the explainable stress prediction model. In order to make sure that these ranges are correct and relevant to medical studies, we collect from the literature reference intervals of features obtained based on experimental studies, which we will refer to as the Ground Truth Data. These ranges are used in the evaluation section to validate the accuracy of our model generated intervals’.

#### Ground truth data collection

We evaluated the results of our stress prediction model using ground truth data collected from experiments that tested the effects of stress on physiological measurements [29–32]. The ground truth data provide information about which physiological features can be used as stress indicators. We compared the list of stress indicators obtained experimentally to the list of features determined by our model to indicate stress.

The previous studies recorded the mean values and standard deviations of features measured during stressful and non-stressful conditions. They then used Kruskal-Wallis tests or Friedman tests to compare mean values between the two conditions to identify statistically significant differences ($p<$0.05). They found that the significant features were $\mu _{NN}$, $\mu _{HR}$, $\sigma _{HR}$, $RMSSD_{HRV}$, $PNN50_{HRV}$, and $\mu _{EDA}$. Table 4 shows the normal ranges, stress ranges, and *p* values of the significant features. In order to extract stress levels of subjects using the EMG signal of the upper trapezius muscle, an experimental procedure was performed in which subjects were faced with three different stressful situations: a calculation task, a logical puzzle task, and a memory task. The EMG signal was found to be a meaningful feature to detect stress, as its amplitude was higher during stress than during relaxed conditions. The same was found for the EMG root mean square values. Therefore, on the basis of the experiments performed, we determined that the following features show elevated EMG amplitude during stressful situations: $\mu _{EMG}$, $RMS_{EMG}$, and $RMS50P_{EMG}$. The respiratory system’s response to stress was reported in [31, 32], showing that the respiration rate $\mu _{RespRate}$ increases during stress.

#### Derivation of the stress ranges and reference intervals

To determine if the measurements are within a non-stressful range, our model provides ranges for each TEST that are related to stress and non-stress, respectively. Such ranges are useful to show what the normal values are for each feature and when the measurements might indicate stressful conditions.

We derive the ranges using the IMPACT values generated for each observation in the training dataset. First, we separate the feature values by their assigned IMPACT values. Then, we group the ones with positive values in a ’Stress Group’ and the ones with negative values in a ’No Stress Group’. We then perform a t-test to make sure that there is a significant difference between the two groups of values. Then, similarly to how many laboratory tests define the Reference Interval, we use a non-parametric approach and take the values falling at the 2.5 and 97.5 percentiles in the No Stress Group as the lower and upper limits of the REFERENCE INTERVAL, respectively. For the ’stress interval’, we use the values falling at the 2.5 and 97.5 percentiles in the Stress Group.

## Experiments and results

We evaluated our explainable AI design for a stress evaluation report through a set of qualitative and quantitative experiments.

The qualitative assessment aimed to determine whether the report provides adequate explanation for the decisions of the AI. In the qualitative assessment, expert psychiatrists were asked the following questions:

1. How useful are the report parameters for the physicians and patients in understanding how the model is making its decision?
2. Does the report provide the AI explanation needed for psychiatrists with examples?
3. What is your opinion concerning to the report’s display and the attached instructions?
4. Can the explainable reports be useful for additional medical applications such as tracking patients’ stress over time or providing other medical insights about the relationships between physiological signals and stress?

The details of the qualitative assessment section are described in “Expert feedback on design of explainable report: a qualitative assessment” section.

The quantitative assessments aimed to evaluate the reliability and accuracy of the following aspects of the explainable AI report, a sample is shown in Fig. 3:

- *STRESS PROBABILITY* To test this aspect, we used a standard ML evaluation approach as described in “Evaluation of the models for stress prediction” section.
- *REFERENCE INTERVAL* To determine how robust the REFERENCE INTERVAL is to changes in the input data, we compared the REFERENCE INTERVALs created using two different subsets of test results, as described in “Evaluation of the ranges and the reference intervals” section.
- *IMPACT* To assess the accuracy of the IMPACT values, we examined the correlations between the IMPACT values and other stress indicators obtained from studies that examined what physiological measurements are affected by stress. The results are described in “Evaluation of the IMPACT” section.
- *FLAGS* To assess the accuracy of the FLAGS as indicators of whether the measurements for a particular factor are indicative of a stressful state, we tested how consistently the two FLAGS for each feature indicated the same stressful state. The results are described in “Evaluation of the FLAGs” section.

The above evaluations were performed using a 4-fold cross validation to ensure balanced subsets of data with sufficient observations. The accuracy of this model, known as the system’s confidence level, is the accuracy of the stress prediction model which can be considered as a historic accuracy based on historic data.

### Expert feedback on design of explainable report: a qualitative assessment

The qualitative assessment aimed to determine whether the psychiatrists and patients can understand the prediction of the AI system and find them useful based on psychiatrists’ opinion. A questionnaire was provided to three expert psychiatrists to provide their evaluation on the explanation report. The questionnaire was accompanied by instructions on how to interpret and read the report in addition to a description of each TEST in the report.

**Fig. 4:**
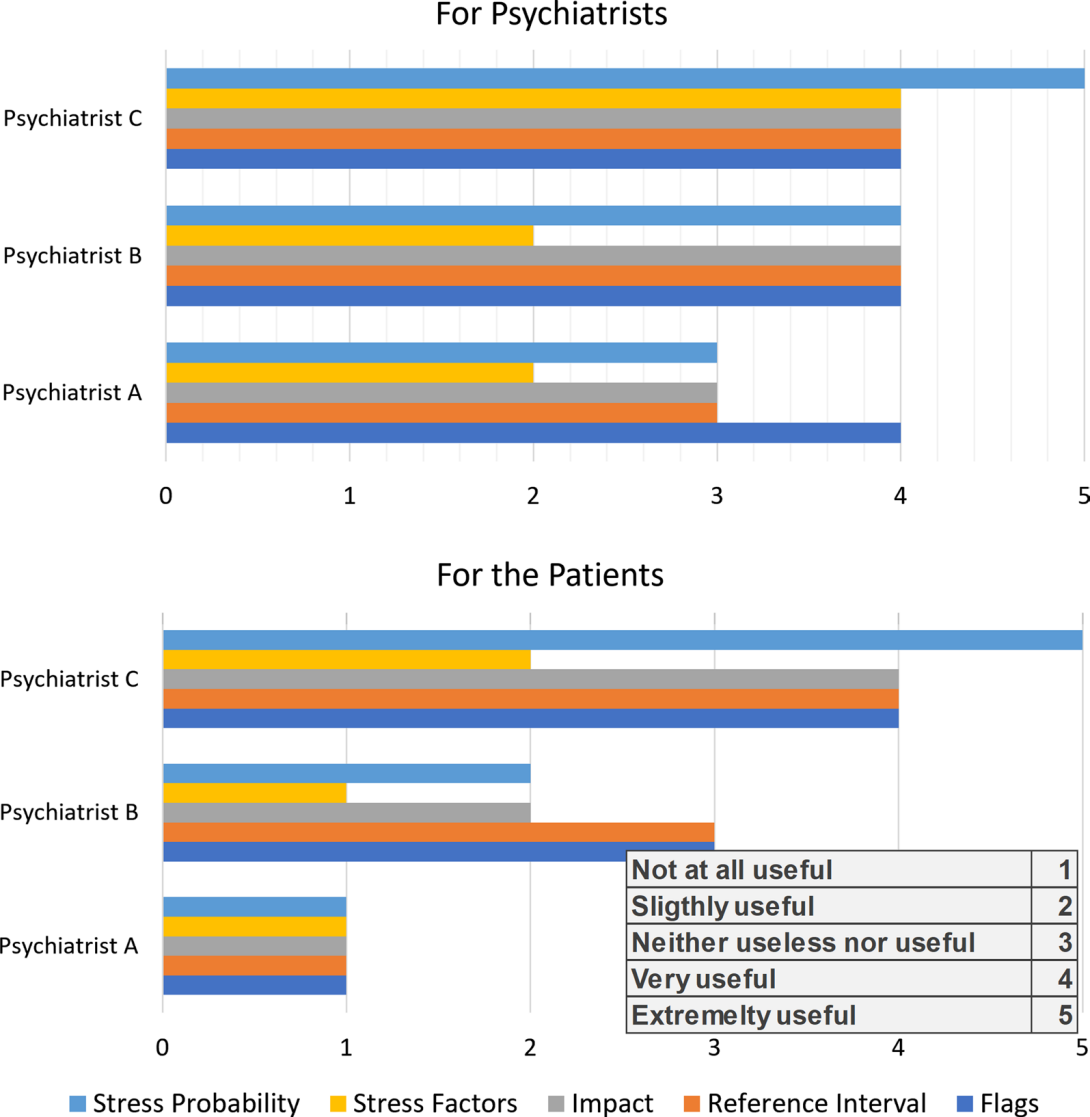
Bar chart listing the usefulness of the report parameters as assessed by three expert psychiatrists

The following section includes a summary of the questionnaire’s result.

1. How useful are the report parameters for the physicians and patients in understanding how the model is making its decision? The expert psychiatrists assessed the report parameters and physiological attributes used to be moderately to extremely useful to help them and the patient understand how the model is making its decision. A bar chart shown in Fig. 4 provides the answers provided by the three psychiatrists.
2. Does the report provide the AI explanation needed for psychiatrists and patients? The psychiatrists agreed that the report does provide the needed explanation for them to understand the AI decision. However, 2 out of 3 psychiatrists thought that this would not be the case for the patients. In addition, the OLAP approach, was found to be moderately important in providing a simpler explanation to the patient.
3. What is your opinion concerning the report’s display and the attached instructions? The psychiatrists found that the report’s display and the instructions manual were easy to follow. However, 2 out of 3 reported that the report’s organization was a little confusing and one found it easy to follow. In addition, 2 out of 3 think that the instructions manual would be slightly clear for the patients.
4. Can the explainable reports be useful for additional medical applications? The expert psychiatrists had different opinions related to the usefulness of the report for a medical diagnosis: ’Not at all’ vs ’Slightly’ vs ’Very Useful’. However, they all reported that the explanation report allows to study the relation between physiological signals and stress. 2 out of 3 found that it could be successfully used to track patient’s stress over time.

### Experiment setup for quantitative evaluation of models: TESTS extraction

We eliminated faulty measurements from the WESAD dataset, such as missing data caused by failures in signal recording. The features listed in Table 2 were extracted from the different physiological raw signals using the numpy, Neurokit [33], and Biosppy [34] libraries in Python. Neurokit is a Python toolbox for statistics and signal processing of data from ECG, EDA, EMG, and EEG. Biosppy is a Python toolbox for bio-signal processing. We extracted data for 42 features, each with 1640 measurements taken over 90-second intervals. The data had an imbalance with 19.7$\%$ stress labels. The $F_{1}$ score was used as the evaluation metric.

### Evaluation of the models for stress prediction

The STRESS PREDICTION is made using the balanced random forest classifier. To evaluate the classifier, the data was divided into four sub-samples and a 4-fold cross-validation approach was followed. Because the dataset was imbalanced, with 19.7$\%$ of the labels representing the class ’stress’, we chose the $F_{1}$ binary score metric, which only reports results for the stress labels. The F1-score obtained from the 4-fold validation are respectively 0.93, 0.63, 0.91 and 0.64. The average $F_{1}$ binary score was 0.78, which is an indication of high accuracy but less than what was achieved in the literature using different input features [8].

### Evaluation of the ranges and the reference intervals

The REFERENCE INTERVAL is defined by the range of values in healthy, non-stressed individuals. The STRESS INTERVAL, on the other hand, includes the test results of stressed individuals. The intervals were determined using the method described in “Derivation of the stress ranges and reference intervals” section. We followed a statistical approach to create the REFERENCE INTERVAL from the No-Stress Group. The 42 features along with their intervals are shown in Table 5 and 6. Features that tend to increase with stress were represented in Table 5, whereas features that were found to decrease with stress are listed in Table 6. We evaluated the REFERENCE INTERVAL by (1) validating that the Stress and No-Stress Groups, separated by the sign of the IMPACT, were independent, belonging to two different distribution and (2) evaluating the robustness of the REFERENCE INTERVAL. To check if the values assigned to the Stress Group and No-Stress Group belonged to two different distributions with two independent ranges, we performed a t-test for each feature in the training dataset. The *p* values obtained for the features are shown in Table 5. For all tests, the *p* value was less than 0.05, which confirmed that the measured values for each feature were significantly different between the stressful condition and the non-stressful condition.

**Table 5:** Intervals and *p* values for the values of each feature under stressful and non-stressful (reference) conditions—where values higher than the reference interval indicate stress

Feature	Stress interval	Reference interval	*p* Value
$\mu _{{ChestEDA}}$	10.73 ± 7.5	3.7 ± 2.95	5E–118
${Min}_{{ChestEDA}}$	11.12 ± 7.84	3.75 ± 3	1E–103
$\sigma _{{ChestEDA}}$	0.15 ± 0.14	0.01 ± 0.01	8E–180
$\mu _{{ChestSCL}}$	− 4.33 ± 4.11	− 14.88 ± 13.52	2E–128
$\mu _{{ChestSCR}}$	15.54 ± 15.3	12.72 ± 11.82	6E–57
$\sigma _{{ChestSCR}}$	5.65 ± 5.47	0.84 ± 0.73	9E–139
${Max}_{{HR}}$	105.74 ± 18.24	73.42 ± 13.06	4E–229
$\mu _{{HR}}$	91.57 ± 15.91	63.79 ± 11.09	7E–229
${Mi}n_{{HR}}$	80.45 ± 14.26	56.6 ± 11.27	5E–215
$\sigma _{{HR}}$	9.04 ± 3.72	3.37 ± 1.85	8E–228
${Max}_{{EMG}}$	1E–02 ± 8E–02	4E-02 ± 3E–02	3E–68
$\mu _{{EMG}}$	1E–07 ± 7E–07	− 1E-07 ± 6E–07	4E–91
${RMS}_{{EMG}}$	1E–02 ± 5E–03	4E-03 ± 9E–04	1E–226
$RMS50P_{{EMG}}$	1E–02 ± 5E–03	4E-03 ± 9E–04	3E–226
$RMS90P_{{EMG}}$	1E–02 ± 5E–03	4E-03 ± 9E–04	3E–225
$\sigma _{{EMG}}$	1E–02 ± 5E–03	4E-03 ± 9E–07	2E–226
$\sigma _{{RespRate}}$	2.08 ± 1.19	0.56 ± 0.42	3E–225
${Max}_{{WristEDA}}$	5.07 ± 4.05	0.55 ± 0.43	4E–223
$\mu _{{WristEDA}}$	4.36 ± 3.69	0.33 ± 0.21	2E–232
${Min}_{{WristEDA}}$	4.23 ± 3.56	0.32 ± 0.21	2E–232
$\sigma _{{WristEDA}}$	0.17 ± 0.16	0.01 ± 0.01	4E–212
$\sigma _{{WristTemp}}$	0.06 ± 0.04	1E–02 ± 4E–03	6E–211

**Table 6:** Intervals and *p* values for the Values of each feature under stressful and non-stressful (reference) conditions—where values lower than the reference interval indicate stress

Feature	Stress interval	Reference interval	*p* Value
${Max}_{{ChestEDA}}$	11.27 ± 7.99	3.79 ± 3.03	1.E–110
$\sigma _{{ChestSCL}}$	1.65 ± 1.5	5.45 ± 4.82	8E–161
$HF_{{HRV}}$	2E+11 ± 2E+11	2E+12 ± 1E+12	5E–227
$LF_{{HRV}}$	3E+11 ± 3E+11	2E+12 ± 2E+12	4E–226
${Mad}_{{NN}}$	21.43 ± 15.72	74.11 ± 42.68	1E–204
${MCV}_{{NN}}$	0.03 ± 0.02	0.09 ± 0.05	4E–196
$|\mu |_{{NN}}$	701.81 ± 82.84	954.91 ± 170.8	1E–219
${Med}_{{NN}}$	693.93 ± 80.36	959.64 ± 191.07	5E–214
${PNN}_{20}$	33.61 ± 28.55	75.35 ± 14.49	1E–220
${PNN}_{50}$	15.15 ± 15.15	38.6 ± 33.12	1E–167
${RMSSD}_{{NN}}$	28.54 ± 18.23	94.8 ± 46.63	2E–230
$\sigma _{{NN}}$	39.89 ± 25.97	107.69 ± 51.22	2E–218
${Min}_{{EMG}}$	− 9E–02 ± 7E–02	− 2E–02 ± 1.E–02	7E–146
#${\ Peaks}_{{EMG}}$	6416.5 ± 866	6955 ± 721	1E–49
${Max}_{{RespRate}}$	13.11 ± 3.36	18.34 ± 2.08	1E–229
$\mu _{{RespRate}}$	11.68 ± 2.69	16.91 ± 2.4	4E–231
${Mi}n_{{RespRate}}$	9.82 ± 2.33	15.32 ± 2.96	7E–232
${Max}_{{WristTemp}},$	31.79 ± 2.12	34.99 ± 0.92	2E–175
$\mu _{{WristTemp}}$	31.69 ± 2.1	34.93 ± 0.93	1E–178
${Min}_{{WristTemp}}$	31.66 ± 2.15	34.91 ± 0.92	5E–173

Because the REFERENCE INTERVAL is obtained using the existing observations, it is dependent on the data used. Therefore, it is important to determine if the range would be different if it were based on another set of observations. We evaluated the robustness by performing again a 4-fold cross validation. In each fold, the REFERENCE INTERVAL from each of the training and testing subsets are generated. We compare these intervals using the relative percentage different (RPD) method which evaluates the change in the REFERENCE INTERVAL. For each feature, we computed the RPD between the intervals generated using the respective subsets with Eq. 6:

6 $$\begin{aligned} RPD_{feature}=\frac{ \vert \mu _{RI_{A}-} \mu _{RI_{B}} \vert }{2 \mu _{RI_{B}}} \end{aligned}$$

By computing the RPD for each feature, we obtained of 16.8$\%$ total difference from the cross-validation as seen in Table 7. between the intervals. Because that difference is relatively small, we concluded that the REFERENCE INTERVAL is robust to changes in the data used to calculate it and is therefore reliable.

**Table 7:** Evaluating the robustness of the reference interval

4-Fold validation	Total RPD ($\%$ )
Fold 1	20.5
Fold 2	15.4
Fold 3	15.4
Fold 4	16.2
Average RPD	16.8

### Evaluation for two key aspects of the report: feature IMPACT and test FLAGs

#### Evaluation of the IMPACT

The IMPACT of each feature was generated using Eqs. 1 and 3, which are based on the SHAP method. The accuracy and success of the SHAP method were proven outside of this paper [8]. The IMPACT can be positive or negative, indicating that the corresponding feature contributes to an increase or decrease in the overall stress probability, respectively. We evaluated the IMPACT parameter from two perspectives: its effectiveness as an indicator of stress and its ability to provide insights into the causes of stress in a given individual.

*Effectiveness of the IMPACT value*

To demonstrate the ability of the IMPACT parameter to explain how each feature affects stress, we examined the correlation between the IMPACT values for the features in our report and the results of previous studies. The previous studies found that the following features were affected by stress: $\mu _{HR}$ , $\sigma _{HR}$ , $RMSSD_{HRV}$ , $PNN50_{HRV}$ , $\mu _{EDA}$ , $\mu _{EMG}$ , $RMS_{EMG}$ , $RMS90P_{EMG},and~ \mu _{RespRate}$ . Those studies recorded for some of the features the range of values that indicated a normal or non-stressful state. For those features, the experimental reference intervals provide insight on whether the test result is indicative of a stressful or normal state. We tested whether the IMPACT parameter could provide the same information by creating a contingency table showing the relationship between the test results that were assigned a positive or negative IMPACT value and the test results that fell within or outside the experimental reference interval. We then performed a Chi-squared test for each feature. We also used the 4-fold cross validation to create a contingency table for each subset of data. A sample of the results of one of the folds is shown in Table 8.

The experiment showed what the normal and stressful ranges were for the ECG and EDA features (Table 4); however, for the EMG features and respiration rate, they only specified if the feature values increased or decreased with stress, without providing normal ranges. Therefore, for those features, the REFERENCE INTERVAL used in the Chi-squared test was the one generated by our model, as shown in Table 5.

We performed the Chi-squared test on each feature of the testing data in each fold with the null hypothesis that the two categories separated on the basis of IMPACT and the REFERENCE INTERVAL were not correlated. A sample of the computed *p* values and the contingency matrix are shown in Table 8. In each of the 4-folds, all of the tests resulted in a *p* value < 0.05, indicating that the null hypothesis was not supported by the data. Therefore, we rejected the null hypothesis and confirmed a correlation between the results of using the REFERENCE INTERVAL and the IMPACT, respectively, as stress indicators. Thus, the IMPACT was found to be an effective parameter to indicate stress.

**Table 8:** Results of chi-squared tests for SHAP evaluation of stress prediction

	Impact	Impact	*p* Value
	> 0	< 0	
${pNN}50_{{HRV}} \notin$ ’Ref. Int.’	5	69	1.76E–07
${pNN}50_{{HRV}} \in$ ’Ref. Int.’	23	12	
${RMSSD}_{{HRV}} \notin$ ’Ref. Int.’	2	57	2.86E–16
${RMSSD}_{{HRV}} \in$ ’Ref. Int.’			
$\mu _{{RespRate}} \notin$ ’Ref. Int.’	72	2	2.86E–25
$\mu _{{RespRate}} \in$ ’Ref. Int.’	0	46	
$\mu _{{WristEDA}} \notin$ ’Ref. Int.’	25	0	2.18E–25
$\mu _{{WristEDA}} \in$ ’Ref. Int.’	1	94	
$\mu _{{WristTemp}}\notin$ ’Ref. Int.’	117	1	3.53E–11
$\mu _{{WristTemp}} \in$ ’Ref. Int.’	0	2	
$\mu _{{NN}}\notin$ ’Ref. Int.’	34	6	0.84E–21
$\mu _{{NN}} \in$ ’Ref. Int.’	0	80	
$\mu _{{HR}}\notin$ ’Ref. Int.’	17	25	1.06E–04
$\mu _{{HR}} \in$ ’Ref. Int.’	7	71	
$\sigma _{{HR}}\notin$ ’Ref. Int.’	39	26	7.60E–06
$\sigma _{{HR}} \in$ ’Ref. Int.’	53	2	
${RMS}_{{EMG}} \notin$ ’Ref. Int.’	81	0	5.80E–26
${RMS}_{{EMG}} \in$ ’Ref. Int.’	53	2	
${RMS50P}_{{EMG}} \notin$ ’Ref. Int.’	80	0	4.75e–25
${RMS50P}_{{EMG}} \in$ ’Ref. Int.’	2	38	
$\mu _{{EMG}} \notin$ ’Ref. Int.’	8	22	1.57E–03
$\mu _{{EMG}} \in$ ’Ref. Int.’	4	86	

*Insights provided by the IMPACT*

**Fig. 5:**
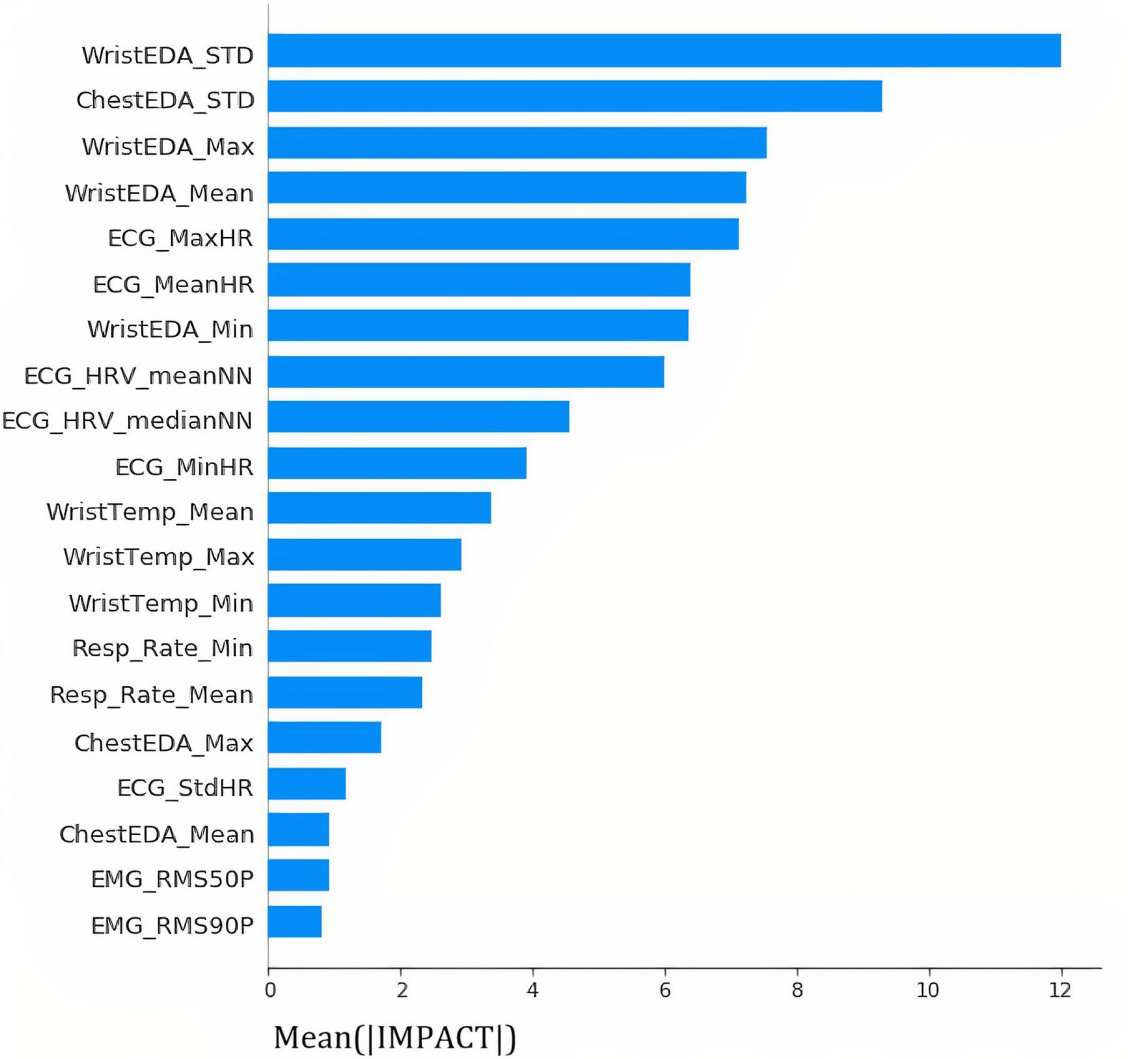
Average impact of physiological features on stress

Figure 5 provides a summary of the mean IMPACT values assigned to each feature from all observations. The length of the bar represents the average impact of the feature on stress. The results show that the main physiological indicators of stress are related to the electrical heart activity and the skin conductance measured from the chest or the wrist.

#### Evaluation of the FLAGs

The two FLAG columns in the stress report inform the patient and health care professionals if any measures should be taken regarding the corresponding feature as it relates to stress. We evaluated the consistency between the two FLAG indicators. Then, we evaluated the insights provided by the FLAGS into the causes of stress.

**Fig. 6:**
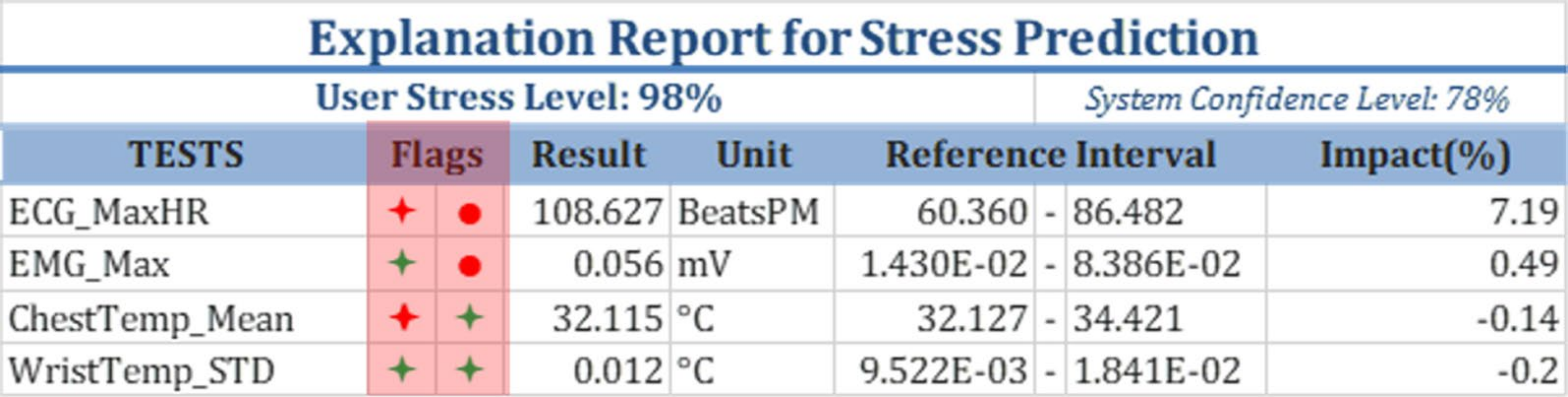
Test results extracted from a sample report

*Consistency between the two FLAGs* We extracted data for four factors from the sample report in Fig. 3 to illustrate the evaluation (Fig. 6). The star-shaped FLAGs are associated with the REFERENCE INTERVAL, whereas the circle-shaped FLAGs are associated with the IMPACT. If the star-shaped FLAG is green, then the measured value of the feature is within the REFERENCE INTERVAL. Red star-shaped FLAGs indicate values that are outside the REFERENCE INTERVAL. If the circle-shaped FLAG is red, then the effect of the feature at the measured level is to increase stress. If the circle-shaped FLAG is green, then the effect of the feature at the measured level is to decrease stress. Because both FLAGS are supposed to indicate signs of stress, they should be consistent for each feature.

**Fig. 7:**
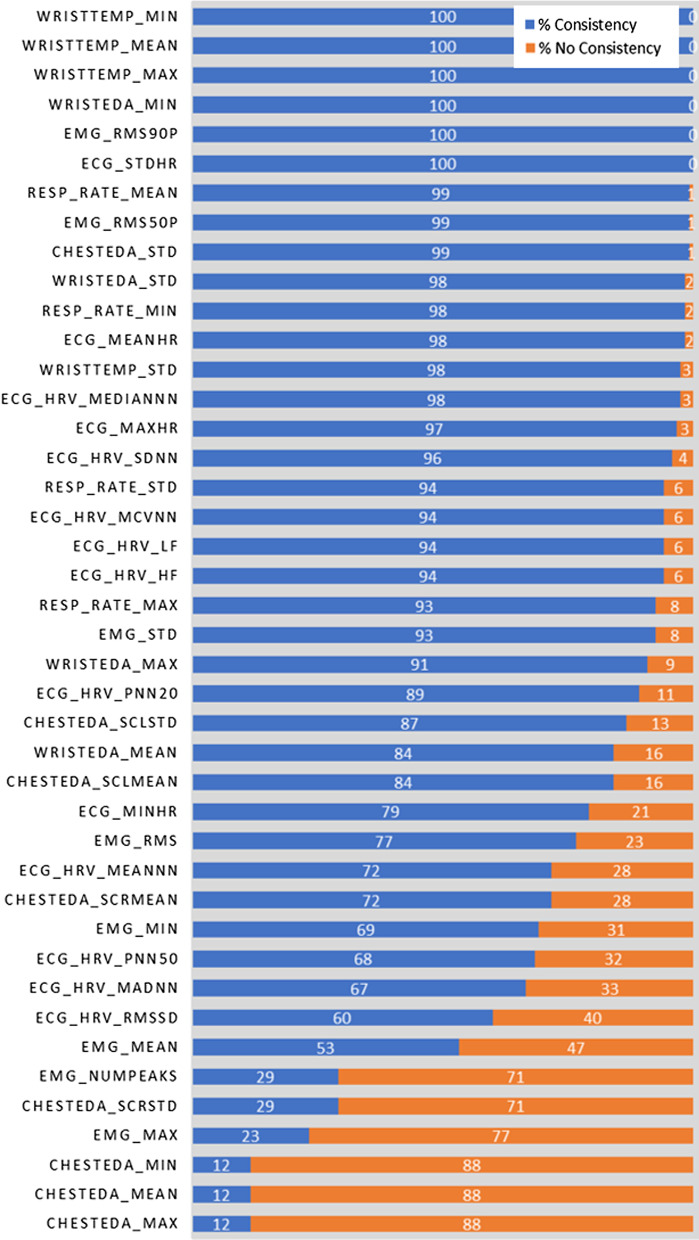
Consistency between the two FLAGS

The FLAGS consistency was again performed by dividing the data into 4 subsets. In each subset, the report was generated per observation of data and the percentage of flag consistency was evaluated. The percentage of consistency per subset of data is represented in Table 9. Overall, there was 80$\%$ consistency between the two FLAGS. The bar charts shown in Fig. 7 show the percentages of reports in the training data which shows the consistency (blue bars) and inconsistency (orange bars) between the FLAGS for each feature. The results showed that the FLAGS with the most inconsistency were mainly associated with the features extracted from the chest EDA and the EMG signal. Four features had inconsistency greater than 50$\%$ . Features with high inconsistency would not be considered good stress indicators compared with other features with low inconsistency.

**Table 9:** Flag evaluation through consistency check

4-Fold validation	Consistency ($\%$ )
Fold 1	81
Fold 2	79
Fold 3	80
Fold 4	80
Average score	80

**Fig. 8:**
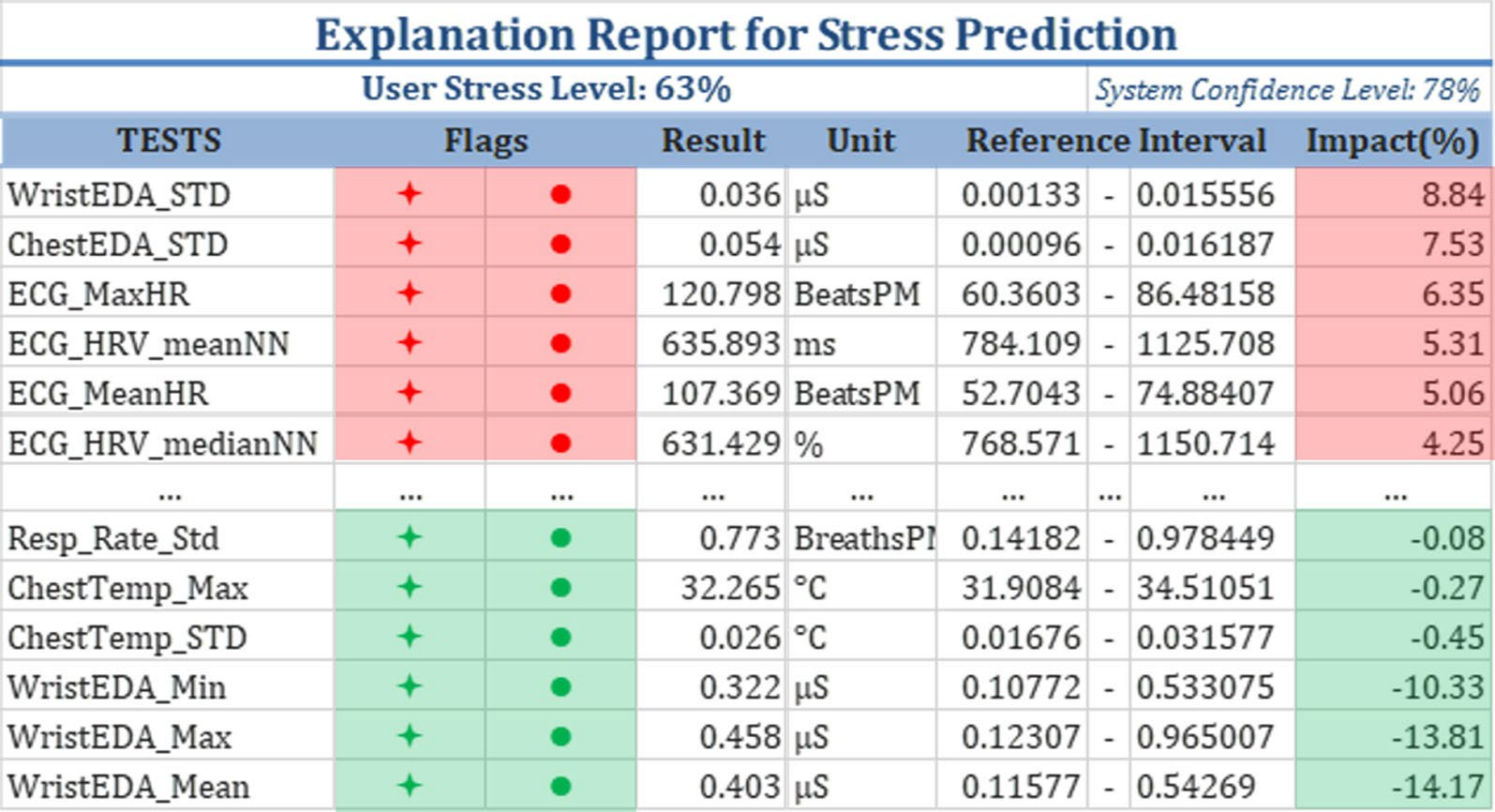
Test Results Extracted from a Sample Report showing insights provided by the FLAGS

*Insights provided by the FLAGS* The FLAGs in the stress prediction report might help explain the predicted stress probability. To illustrate that, we consider an example report generated for one individual (Fig. 8). In that report, the model predicted that the user was stressed with a probability of 63$\%$ . The values registered for the ECG signal and chest EDA indicate a stressful state, which is represented by the positive IMPACT and the red FLAGS. The 37$\%$ model uncertainty is due to the features that had green FLAG indicators, which include the minimum, maximum, and mean values of the EDA signal recorded from the wrist.

## Discussion

In this section, we discuss the additional analysis that could be extracted on how stress could have a different effect on physiological measurements based on the person’s age and gender. We also discuss one of the limitations, the small dataset, and our proposed future work to overcome it.

### Discussion on difference in reference intervals based on age and gender

The Reference Interval per physiological measurement, indicating the no-stress range, might be different between genders and more specifically it might be different per individual. In this section, we aim to study the difference in reference intervals generated by gender and then per individual for some features. For this analysis, we will consider the features that were assigned a positive impact higher than 5 $\%$ in the report of Fig. 3. These features are: $\sigma _{ChestEDA}$ , $\sigma _{WristEDA}$ , $Max_{HR}$ , $\mu _{WristEDA}, Max_{WristEDA}$ , $\vert \mu \vert _{NN}$,and $\mu _{HR}$. We aim to study if a significant difference is found between the reference intervals generated:

- Based on gender.
- Per individual.

By generating the reference interval using the data of each individual separately, we found that the reference intervals of $\sigma _{Wrist_{EDA}}$,$\sigma _{Chest_{EDA}}$ and $Max_{HR}$ showed the higher difference between individuals, compared to the other studied features.

However, if we compare the reference intervals of the same features by separating the subjects into Males and Females, we found that the main difference in reference intervals was in the $\vert \mu \vert _{NN}$, $\mu _{HR}$ and $Max_{HR}$. However since the data was collected from 3 Females and 11 Males and since we have few inputs per individual, we cannot confirm our analysis as a larger dataset is required to draw much reliable insights.

### Discussion on the “best” experiment for a better dataset

The main limitation of this work is the relatively small dataset used which was caused by faulty measurements and small number of participants. In addition, we have no wide age range since the participants from which the WESAD data was collected from were graduate students. Therefore, in this section we explain what would be the optimal experiment to perform to collect the data needed to get more accurate and stable analysis to obtain the following:

(1) More robust reference intervals following the evaluation done in “Evaluation of the ranges and the reference intervals” section. (2) More accurate analysis on the difference of reference intervals based on Age and Gender as discussed in “Discussion on difference inreference intervals based on age and gender” section.

Our future work therefore includes the implementation of a user-study that takes into consideration all the missing parameters that would have been useful in our analysis, a larger dataset. For the proposed user-study, the number of participants should be around 50 subjects, separated between 25 Males and 25 Females. We would separate them into 4 groups to compare between the normal and stress related physiological measurements based on gender and age : (1) Females between 18–25, (2) Females between 26–35, (3) Males between 18–25 and (4) Males between 26–35. The collected measurements will include the Respiration Signal, the ECG signal, the EMG signal collected from the Trapezius muscle and the EDA and Temperature measured from the wrist. Two different experiments can be performed. The first experiment would be in a controlled environment which includes a series of relaxed and stressful tasks to be performed by the 50 subjects. For the stress conditions, the users will be exposed to the trier social stress test (TSST) as well as puzzles and logical tasks. The duration of the stress experiments will be 2 hours. As for the relaxed conditions, the subjects will be provided neutral reading materials such as magazines for 20 min, they will be required to watch a set of funny video clips for 15 min, for amusement and finally they will perform controlled breathing exercises after each stress experiment in the aim of returning to a close to neutral/no stress state. The duration is 7 min and to be performed after each stress experiment. The labels will be collected using three self-reports: PANAS, STAI (State-Trait Anxiety Inventory) and Short Stress State Questionnaire (SSSQ). Another type of experiment can be performed to collect the needed data in the “wild”. The users will be asked to collect around 10 hours of measurements. More data is required in this experiment to make sure we have enough stress labels and since in the “wild” there are many factors that could lead to the collection of incorrect/faulty measurements (such as disconnected device, low battery, ...), every 45 min the user will be asked to fill out the PANAS and the STAI questionnaires.

## Conclusion

In this work, we provided a new design for explainable AI used in stress prediction based on physiological measurements. To make AI-based stress evaluation more user-friendly and medically beneficial, the report is configurable on the basis of users’ needs. Based on the report, users can determine what biological features have the most impact on the prediction of stress in addition to any health-related abnormalities.

We developed AI models that can produce the necessary explanations. The physiological measurements used in the stress report include signals related to heart activity, muscle activity, body temperature, and skin conductance. The report uses the same physiological features that are commonly used in experiments to study the biological effects of stress.

The effectiveness of the report was evaluated using a quantitative and a qualitative assessment. The stress prediction accuracy was shown to be comparable to state of the art at an F1-score of 0.78. The contributions of each physiological signal to the stress prediction was shown to correlate with ground truth. The evaluation of the reference interval showed that the chosen intervals were reliable. In addition to these quantitative evaluations, a qualitative survey with psychiatrists confirmed the clinical usefulness of the explanation report as generated by the AI system. Future work should include the addition of more explanatory features related to specific emotional states of the patients, such as sadness, anxiety, and happiness. In addition to the implementation of a user-study to collect a larger dataset. This dataset will allow separating the data of individuals based on gender and age group and obtain enough observations per user for a better analysis and more accurate results. Finally, focus group discussions and in-depth interviews of users and psychiatrists would be performed as future work to explain the results provided by our stress explanation report and optimize our work accordingly.

## Data Availability

The dataset used in this work is the WESAD (Wearable Stress and Affect Detection) dataset. This dataset is a public dataset and can be downloaded from https://ubicomp.eti.uni-siegen.de/home/datasets/icmi18/.

## Footnotes

1 http://www.biosignalsplux.com/en/respiban-professional.

2 http://www.empatica.com/research/e4/.
